# A Comparative Review of Performance and Concurrent Cardiac Rhythm Assessment Across Home Sleep Apnea Testing Devices

**DOI:** 10.7759/cureus.118688

**Published:** 2026-10-27

**Authors:** Brian Brockway, Arjun Sharma, Clara Stein, Sarthak Jain, Marina Brockway

**Affiliations:** 1 Development, Rhythm Express, Saint Paul, USA; 2 Cardiology, Rhythm Express, Reno, USA

**Keywords:** apnea-hypopnea index, atrial fibrillation burden, home sleep apnea testing, obstructive sleep apnea, sleep-disordered breathing

## Abstract

Home sleep apnea testing (HSAT) is now widely used in place of in-laboratory polysomnography (PSG) for the evaluation of sleep-disordered breathing (SDB), but published performance varies across FDA-cleared devices. This narrative review aimed to summarize the published performance of FDA-cleared Type III HSAT devices currently marketed in the United States and compare their labeled capabilities for assessing SDB over multiple nights and concurrently evaluating cardiac rhythm and atrial fibrillation (AF) burden. A search of the FDA 510(k) database identified 68 devices with MNR as the primary product code that were cleared after August 1, 2016, or were cleared earlier but released for sale after that date.

There were 13 devices that met these criteria and the Centers for Medicare and Medicaid Services (CMS) criteria for a Type III HSAT. For each device, sensitivity and specificity against PSG, scored under American Academy of Sleep Medicine Rule 1B (4% oxygen desaturation), were obtained from the FDA 510(k) Summary or a peer-reviewed validation study. Balanced accuracy (the mean of sensitivity and specificity) was calculated at apnea-hypopnea index (AHI) thresholds of 5, 15, and 30 events per hour. Five devices were excluded from the comparison and are reported for information only because their performance data were not reported using a methodology consistent with that of the other devices: they used a different scoring rule or severity threshold, reported central apnea detection only, or relied on a less credible source for the reported data.

Among the remaining eight devices, reported sample sizes ranged from 30 to 476 subjects. All eight reported performance at AHI ≥ 15, five at AHI ≥ 5, and three at AHI ≥ 30. At AHI ≥ 15, balanced accuracy was highest for AcuPebble Ox200 (Acurable Limited, London, UK) (96.5), followed by WatchPAT SW (Itamar Medical, Caesarea, Israel) (94.0) and RX-1 Sleep (Rhythm Express, St. Paul, MN) (92.2). RX-1 Sleep reported the highest balanced accuracy at AHI ≥ 5 (90.9) and AHI ≥ 30 (92.9). These comparisons are indirect and descriptive: each device was validated against PSG in a separate cohort, none was evaluated against another device in the same patients, and between-device differences were not statistically tested. Prospective head-to-head studies against a common reference standard would be needed to confirm differences in relative performance.

Among the devices compared, RX-1 Sleep system provides an aid for concurrent assessment of SDB and AF burden in a single test. Other devices can flag or record rhythm information but cannot quantify AF burden. Together with its published performance and multi-night testing capability, this may offer clinical benefit given the severity-dependent association between SDB and AF burden and the elevated cardiovascular risk observed when the two conditions coexist.

## Introduction and background

Sleep-disordered breathing (SDB), including obstructive sleep apnea (OSA) and central sleep apnea (CSA), is highly prevalent among adults and can have serious consequences for health and quality of life [1]. Early diagnosis and management are important for improving patient outcomes and reducing associated healthcare costs [2]. Despite its clinical significance, SDB remains underdiagnosed, in part because of the financial and logistical barriers associated with in-lab polysomnography (PSG) [3].

PSG is considered the clinical standard for diagnosing sleep disorders but requires patients to undergo an overnight study in a specialized facility where trained personnel monitor multiple physiological signals. Although PSG provides a comprehensive assessment of sleep and breathing, it is expensive, inconvenient, and uncomfortable for patients. Overnight recording in an unfamiliar laboratory environment can often disrupt sleep and may influence study results [4]. Home sleep apnea testing (HSAT) provides a more accessible and convenient alternative for appropriately selected patients. The American Academy of Sleep Medicine (AASM) recommends the use of technically adequate HSAT devices for diagnosing OSA in uncomplicated adults at increased risk of moderate-to-severe OSA; these devices are typically classified as Type III by the Centers for Medicare and Medicaid Services (CMS) [5]. Following diagnosis, home sleep testing may also be used in selected circumstances to assess treatment response [6].

The use of HSAT has grown substantially in recent years, accompanied by the development of new devices and technologies intended to expand access to sleep testing; among Medicare beneficiaries, home testing has progressively displaced in-laboratory PSG as the predominant diagnostic modality [7]. Compared with PSG, HSAT systems typically use fewer sensors to improve portability, usability, and patient comfort. Testing can be performed in the patient’s home, in their own bed, and may be less disruptive to normal sleep, potentially improving patient acceptance and adherence. Type III HSAT devices record four to seven signals [1,8], providing a practical balance between diagnostic information, cost, and patient convenience.

The reduced number of physiological signals, however, can limit published performance relative to PSG and may affect the accuracy of sleep apnea severity classification [9,10]. Conversely, more comprehensive HSAT systems use sensors such as respiratory effort belts and nasal cannulas that can be cumbersome for patients to apply and maintain during an unsupervised overnight study. Thus, existing HSAT technologies involve an inherent tradeoff between the amount of physiological information collected, performance, and patient usability.

High performance is important for minimizing underdiagnosis, misclassification, and unnecessary repeat testing [11-13]. HSAT performance is evaluated by comparing HSAT-derived values of the apnea-hypopnea index (AHI), classified as mild (5 to < 15 events/h), moderate (15 to < 30 events/h), or severe (≥ 30 events/h), with those obtained by PSG, using sensitivity and specificity at these thresholds [1]. Hypopnea scoring depends on the oxygen desaturation threshold applied, and the AASM accepts either ≥ 3% from baseline (Rule 1A) or ≥ 4% (Rule 1B) [14]. Since reported performance differs according to which rule is used, all comparisons in this review are based on Rule 1B, the criterion for which data were most consistently available across devices.

One physiological signal routinely acquired during PSG but absent from many HSAT systems is the ECG. PSG primarily relies on electroencephalography and electrooculography to characterize sleep and determine total sleep time, while ECG provides information regarding heart rate, rhythm, and cardiovascular disturbances occurring during sleep [15]. Because most HSAT systems do not incorporate electroencephalography or electrooculography, sleep/wake and sleep-stage estimates may instead be derived from combinations of signals such as peripheral arterial tone, oxygen saturation, body movement, and heart rate [9]. Incorporating ECG provides an additional physiological signal that may contribute to sleep/wake or sleep-stage estimation while simultaneously providing information about cardiac rhythm during sleep [16-18].

The ECG is particularly relevant because SDB is closely associated with rhythm disturbances, atrial fibrillation (AF) in particular. Recurrent hypoxemia, autonomic activation, and inflammation associated with sleep apnea can increase cardiovascular stress and have been associated with hypertension, cardiac arrhythmias, heart failure, and sudden cardiac death [19], and OSA has specifically been associated with an increased risk of major adverse cardiovascular and neurologic events among patients with pre-existing AF [20,21]. ECG acquired during an HSAT may therefore identify clinically relevant rhythm disturbances that warrant additional cardiovascular evaluation.

ECG also provides beat-to-beat heart-rate information from which heart-rate variability and other autonomic features associated with sleep apnea severity [22] can be derived. Apneas and hypopneas produce characteristic autonomic responses, including cyclic changes in heart rate, that can provide an additional signal for identifying respiratory events [23,24]. ECG may therefore complement respiratory and oximetry signals while simultaneously providing information regarding cardiac rhythm and autonomic responses associated with SDB. Because SDB severity varies from night to night, and because that variability has been linked to AF burden, concurrent assessment of SDB and cardiac rhythm across multiple nights has been proposed as a means of characterizing the relationship between the two [25]; this rationale and its limitations are considered in the Discussion section.

The devices included in this review differ in the physiological signal ensemble they record, the number of nights they are used to aid in the assessment of, and whether they provide cardiac rhythm information; these differences are compared in the Results section. The objective of this narrative review is to summarize the published performance of FDA-cleared, CMS Type III HSAT devices currently marketed in the United States, and to compare their labeled capabilities for multi-night sleep apnea assessment and for concurrent cardiac rhythm and AF-burden monitoring. Devices were identified from the FDA 510(k) database and were included according to the prespecified eligibility criteria described in the Methods section. Devices were not selected on the basis of market share, for which device-level utilization data are not publicly available, and no device was treated as an index device against which the others were compared.

## Review

Methods

This analysis is presented as a narrative, comparative review of publicly available performance data rather than a formal systematic review; it does not follow PRISMA (Preferred Reporting Items for Systematic Reviews and Meta-Analyses) reporting guidelines and was not based on a registered review protocol. Where diagnostic-accuracy data were drawn from heterogeneous sources of varying study design, sample size, and population, this is noted explicitly later on and addressed as a limitation in the Discussion section. The review summarizes published performance across all devices meeting the eligibility criteria below; no device was designated in advance as an index device or as a comparator.

A search was conducted to identify FDA-cleared devices actively marketed for unattended home assessment of SDB in patients suspected of having sleep apnea. Each identified device was then independently screened by two authors to confirm that it meets the CMS criteria for a Type III device for evaluating SDB, that a credible source of evidence exists for its accuracy in detecting sleep apnea severity, and that it is currently marketed in the United States.

Devices were identified by searching the U.S. FDA 510(k) CDRH database for Traditional 510(k) clearances (https://www.accessdata.fda.gov/scripts/cdrh/cfdocs/cfpmn/pmn.cfm) with primary product code MNR that were either granted within the preceding 10 years (since August 1, 2016), or granted before that date and released for sale at a later time. Product code MNR denotes Ventilatory Effort Recorders, a category that encompasses devices intended for unattended assessment of SDB in the home without supervision by a health care provider. MNR is also used as a secondary product code on other devices, including CPAP machines and ventilators as well as portable PSG systems classified by CMS as Type II devices for evaluating SDB; these were not eligible for inclusion in this comparison.

Each device returned by the query was additionally required to satisfy all the following: (1) meeting the CMS criteria for a Type III sleep test for obstructive sleep apnea, recording four to seven signals, for unattended home use; (2) reporting sensitivity and specificity against polysomnography as the reference standard for at least one sleep apnea severity level, with those results published in the device’s FDA 510(k) Summary or in a credible peer-reviewed publication; (3) apnea-hypopnea events detected using AASM Rule 1B; and (4) marketed in the United States as of August 1, 2026.

Sixty-eight devices had MNR as their primary product code and were either cleared since August 1, 2016, or cleared before that date and released for sale after it. After excluding devices classified by CMS as Type II or Type IV, devices requiring attended monitoring or physician supervision, and devices no longer marketed in the United States, 13 devices remained and are compared here.

A formal risk-of-bias instrument was not applied to the underlying sources. Instead, limitations identified in each device’s source of performance data - non-standard severity thresholds, scoring rules that could not be confirmed, manually scored results only, data reported under AASM Rule 1A rather than Rule 1B, performance reported for central apnea detection only, performance cited in another manufacturer’s 510(k) Summary rather than in a peer-reviewed publication, and results available only as a conference abstract - are recorded in the review. Devices carrying such a limitation are reported for information only and are excluded from the between-device comparison of balanced accuracy, because their published estimates were not obtained on a basis comparable with the remaining devices. Their values are retained in a table so that the publicly available evidence remains visible without being read as directly comparable.

In addition to the quantitative accuracy comparison, FDA 510(k) Premarket Notices and manufacturer materials for each included device were reviewed to compare labeled capability for multi-night SDB testing and for cardiac rhythm and AF monitoring. For each device, we recorded the sensitivity and specificity against PSG reported at each AHI threshold. Values were taken from the device’s own FDA 510(k) Summary where available, and otherwise from a validation study identified by searching PubMed for the device name and manufacturer. Hypopneas may be scored using a ≥ 3% oxygen desaturation from baseline (AASM Rule 1A) or the stricter ≥ 4% (Rule 1B) [14]; reported performance differs between the two, so Rule 1B was used throughout. Study designs were reviewed to confirm that the reported data corresponded to Rule 1B scoring. Where the only identifiable validation was of a predicate or earlier iteration of a currently marketed device, data in the table indicate this explicitly, as substantial equivalence for regulatory purposes does not establish that the marketed device achieves the same accuracy.

Balanced accuracy - the average of sensitivity and specificity - was used to summarize performance at each threshold, weighting the two equally. A pooled receiver operating characteristic (ROC) analysis [26] was not possible because device-level ROC curves and confidence intervals (CIs) are not available for most devices. Balanced accuracy at a fixed threshold is therefore a simple summary of published point estimates rather than a statistically weighted comparison, and differences between devices should be read as descriptive rather than as evidence of superiority. Each device’s sensitivity and specificity at a given threshold are plotted as a single point against contours of constant balanced accuracy.

Results

The search identified 68 devices with MNR as the primary product code that were cleared after August 1, 2016, or cleared earlier and released for sale after that date. Applying the selection criteria, which excluded devices classified by CMS as Type II or Type IV, devices requiring attended monitoring or physician supervision, and devices no longer marketed in the United States, left 13 devices for comparison. Where a manufacturer had obtained multiple clearances for successive iterations of the same platform, the device associated with the most recent clearance was used.

Thirteen devices met the selection criteria and were evaluated in detail through review of FDA 510(k) Premarket Notices, manufacturer materials and, where applicable, peer-reviewed validation studies. They are listed in decreasing order of 510(k) number in Table 1. Two FDA clearances of SANSA HSAT (Huxley Medical, Atlanta, GA) are listed separately because they report different performance claims: K240285 reports obstructive apnea performance and K252497 reports central apnea detection. Where available, the number of subjects underlying each device’s reported performance is given in Table 1. Reported sample sizes ranged from 30 to 476 subjects, could not be confirmed for WatchPAT SW (Itamar Medical, Caesarea, Israel), and are not attributable to WatchPAT 400 (Itamar Medical), whose figures are pooled across studies. Device-specific limitations in the underlying evidence are reported in the final column of Table 1 and are discussed below.

**Table 1:** Performance of the included FDA-cleared Type III HSAT devices against polysomnography under AASM Rule 1B (≥4% oxygen desaturation) N/A indicates that the source did not report performance at that threshold FDA: the Food and Drug Administration; HSAT: home sleep apnea testing; AASM: the American Academy of Sleep Medicine; AHI: apnea-hypopnea index; CI: confidence interval; SE: sensitivity; SP: specificity

Product; manufacturer; 510(k) number	Source	AHI threshold	Sensitivity (%)	Specificity (%)	95% CI	Balanced accuracy	Study size	Limitations
WatchPAT SW (Itamar Medical); K254042	K254042 [27]	≥ 5	N/A	N/A	N/A	N/A	Not reported	—
		≥ 15	92	96	N/A	94		
		≥ 30	N/A	N/A	N/A	N/A		
RX-1 Sleep (Rhythm Express); K253148	K253148 [28]	≥ 5	87.8	93.91	SE: (83.05, 91.62); SP: (90.00, 96.63)	90.9	476	—
		≥ 15	88.54	95.79	SE: (80.42, 94.14); SP: (93.25, 97.57)	92.2		
		≥ 30	88.24	97.51	SE: (72.55, 96.70); SP: 95.59, 98.75)	92.9		
SANSA HSAT (Huxley Medical); K252497	K252497 [29]	≥ 5	N/A	N/A	N/A	N/A	325	Central sleep apnea detection only. Non-standard AHI severity threshold. Rule is not clear
		≥ 10	100	98.7	SE: (73.5, 100); SP: (96.8, 99.)	99.4		
		≥ 30	N/A	N/A	N/A	N/A		
WatchPAT 400 (Itamar Medical); K250460	Iftikhar et al., 2022 [30]	≥ 5	94.11	43.47	N/A	68.8	Pooled analysis	—
		≥ 15	92.21	72.39	N/A	82.3		
		≥ 30	74.11	87.1	N/A	80.6		
AcuPebble Ox200 (Acurable Limited); K243092	Devani et al., 2021 [31]	≥ 5	N/A	N/A	N/A	N/A	150	—
		≥ 15	95.92	97.03	N/A	96.5		
		≥ 30	N/A	N/A	N/A	N/A		
SANSA HSAT (Huxley Medical); K240285 [32]	Goldstein et al., 2025 [33]	≥ 5	94	71	SE: (90, 96); SP: (62, 79)	82.5	340	—
		≥ 15	88	87	SE: (81, 93); SP: (82, 91)	87.5		
		≥ 30	82	95	SE: (71, 90); SP: (92, 97)	88.5		
SAM Model 9-10000 (Snap Diagnostics); K240100	Michaelson et al., 2006 [34]	≥ 5	59	96	N/A	77.5	59	—
		≥ 15	38	100	N/A	69		
		≥ 30	N/A	N/A	N/A	N/A		
Wesper (Wesper Lab); K221816 [35]	Rohrscheib et al., 2026 [36]	≥ 5	90	60	N/A	75	45	—
		≥ 15	67	86	N/A	76.5		
		≥ 30	N/A	N/A	N/A	N/A		
ANNE Sleep (Sibel Health, Inc.); K220095	Davies et al., 2022 [37]	≥ 5	96	86	N/A	91	225	Manual scoring results only
		≥ 15	90	97	N/A	93.5		
		≥ 30	65	99	N/A	82		
SomnaPatch (Respironics, Inc.); K183625	Merchant et al. [38]	≥ 5	N/A	N/A	N/A	N/A	174	Conference abstract
		≥ 15	86	83	N/A	84.5		
		≥ 30	N/A	N/A	N/A	N/A		
SleepImage (MyCardio, LLC); K182618	Performance cited in another manufacturer’s 510(k) Summary	≥ 5	98.7	84.8	SE: (97, 100); SP: (72.6, 97.1)	91.8	189	Performance cited in Ensodata 510K summary, not a peer-reviewed publication. Reported for Rule 1A only
		≥ 15	N/A	N/A	N/A	N/A		
		≥ 30	N/A	N/A	N/A	N/A		
Apnea Risk Evaluation System (ARES), Model 620 (Watermark Medical); K160499	Westbrook et al., 2005 [39]	≥ 5	N/A	N/A	N/A	N/A	284	Non-standard AHI severity threshold. Rule is not clear
		≥ 10	85	91	N/A	88		
		≥ 30	N/A	N/A	N/A	N/A		
NOX T3s (Nox Medical); K082113. Launched in 2020	Delaet et al., 2026 [40]	≥ 5	N/A	N/A	N/A	N/A	30	—
		≥ 15	77.8	81.8	N/A	79.8		
		≥ 30	N/A	N/A	N/A	N/A		

Five devices carried an identified limitation in their source of performance data, recorded in the final column of Table 1. Because their performance was not obtained on a basis comparable with the remaining eight, they do not enter the ranking below. Their values are retained in Table 1 for information because they represent the publicly available evidence for devices that otherwise meet the selection criteria.

SANSA HSAT clearance K252497 reports performance for central apnea detection only, at a non-standard threshold of AHI ≥ 10 and under a scoring rule that could not be confirmed; ANNE Sleep (Sibel Health, Inc., Chicago, IL) reports manually scored results only; SleepImage System (MyCardio, LLC, Broomfield, CO) performance is cited in another manufacturer’s 510(k) Summary rather than a peer-reviewed publication and is scored under AASM Rule 1A; Apnea Risk Evaluation System (ARES) Model 620 (Watermark Medical, West Palm Beach, FL) reports performance only at a non-standard threshold of AHI ≥ 10, under a scoring rule that could not be confirmed; and SomnaPatch (Respironics, Inc., Murrysville, PA) performance data are available only as a conference abstract.

Balanced accuracy for the eight devices that met all criteria is ranked below at each threshold. A device appears under a threshold only where its source reported performance at that threshold.

AHI ≥ 5** **(Five Devices)

RX-1 Sleep (Rhythm Express, St. Paul, MN): 90.9; SANSA HSAT: 82.5; SAM Model 9-10000 (Snap Diagnostics, Vernon Hill, IL): 77.5; Wesper Lab (New York, NY): 75.0; WatchPAT 400: 68.8. 

AHI ≥ 15 (Eight Devices)

AcuPebble Ox200 (Acurable Limited, London, UK): 96.5; WatchPAT SW: 94.0; RX-1 Sleep: 92.2; SANSA HSAT: 87.5; WatchPAT 400: 82.3; NOX T3s (Nox Medical, Reykjavík, Iceland): 79.8; Wesper Lab: 76.5; SAM Model 9-10000: 69.0.

AHI ≥ 30 (Three Devices)

RX-1 Sleep: 92.9; SANSA HSAT: 88.5; WatchPAT 400: 80.6.

Because the estimates derive from different studies, populations, and reporting standards, and because only the AHI ≥ 15 threshold is supported by all eight devices, this ordering is descriptive, was not statistically tested, and should be interpreted with particular caution at AHI ≥ 5 and ≥ 30.

The HSAT devices vary in their ability to analyze and report cardiac arrhythmia. Of the 13 devices reviewed, only the RX-1 Sleep system, utilizing RX-1 mini measurements, is capable of concurrent evaluation of SDB and AF burden in a single test. RX-1 Sleep is a Software as a Medical Device cleared for the diagnostic evaluation of sleep-related disordered breathing in adults [28]; it analyzes data acquired from the RX-1 mini two-channel ECG monitor and the Checkme O2 Ultra wrist-worn pulse oximeter (Viatom, Shenzhen, China) using a cloud-based algorithm and is labeled as an aid in assessment of SDB across multiple nights. RX-1 mini is FDA cleared (K241179) to aid in the assessment of AF burden and other arrhythmias for up to 30 days. SANSA HSAT received FDA clearance to record and store up to 10 hours of ECG data per night for up to two nights for later analysis as an aid in providing an arrhythmia diagnosis [41] (K250882). This recording duration is generally not considered suitable for aiding in the assessment of AF burden.

SANSA HSAT received FDA clearance to record and store ECG data that can later be manually analyzed to inform an arrhythmia diagnosis [41]. However, the device records ECG for “Approx. 10 hours per study, two nights of study maximum” (K250882), a duration not suitable for assessing AF burden. WatchPAT 400 can flag “suspected atrial fibrillation” and premature beats but is not diagnostic [42]. Similarly, NOX T3s can detect atrial fibrillation only if an additional ECG channel is added and activated during a sleep study, and these findings are not diagnostic [43]; thus, AF burden cannot be assessed. This comparison does not reflect a head-to-head measurement of AF detection accuracy across devices due to the varying labeled device capabilities.

Discussion

*Performance* *at Clinically Decisive Thresholds*

Accurate diagnosis of sleep-disordered breathing is essential to managing the condition effectively, and to the health and cost consequences that follow from it [16]. The AASM recommends HSAT for patients with a high pre-test probability of moderate-to-severe OSA rather than for those suspected of mild disease, because HSAT is less sensitive than PSG [5]. That recommendation places the diagnostic burden squarely on performance at AHI ≥ 15 and AHI ≥ 30 - the thresholds separating moderate and severe disease, and the ones that most often determine treatment eligibility. Balanced accuracy is a useful summary at these thresholds because it weights the two kinds of error equally, and the two kinds of error carry real but different costs: a false negative leaves significant disease untreated, while a false positive commits a patient to therapy, cost, and follow-up they do not need.

Measured against that standard, the published evidence is uneven. Of the 13 devices meeting the selection criteria, five could not be included for comparison because their performance data were not obtained on a comparable basis. Among the eight that could be compared, every device reports performance at AHI ≥ 15, but only five report at AHI ≥ 5 and only three at AHI ≥ 30. At AHI ≥ 15, the highest balanced accuracy was reported by AcuPebble Ox200 (96.5), WatchPAT SW (94.0), and RX-1 Sleep (92.2), and the spread across the eight devices was wide, from 96.5 down to 69.0. At AHI ≥ 30, only RX-1 Sleep (92.9), SANSA HSAT (88.5) and WatchPAT 400 (80.6) reported data at all (Figures 1-3).

**Figure 1:**
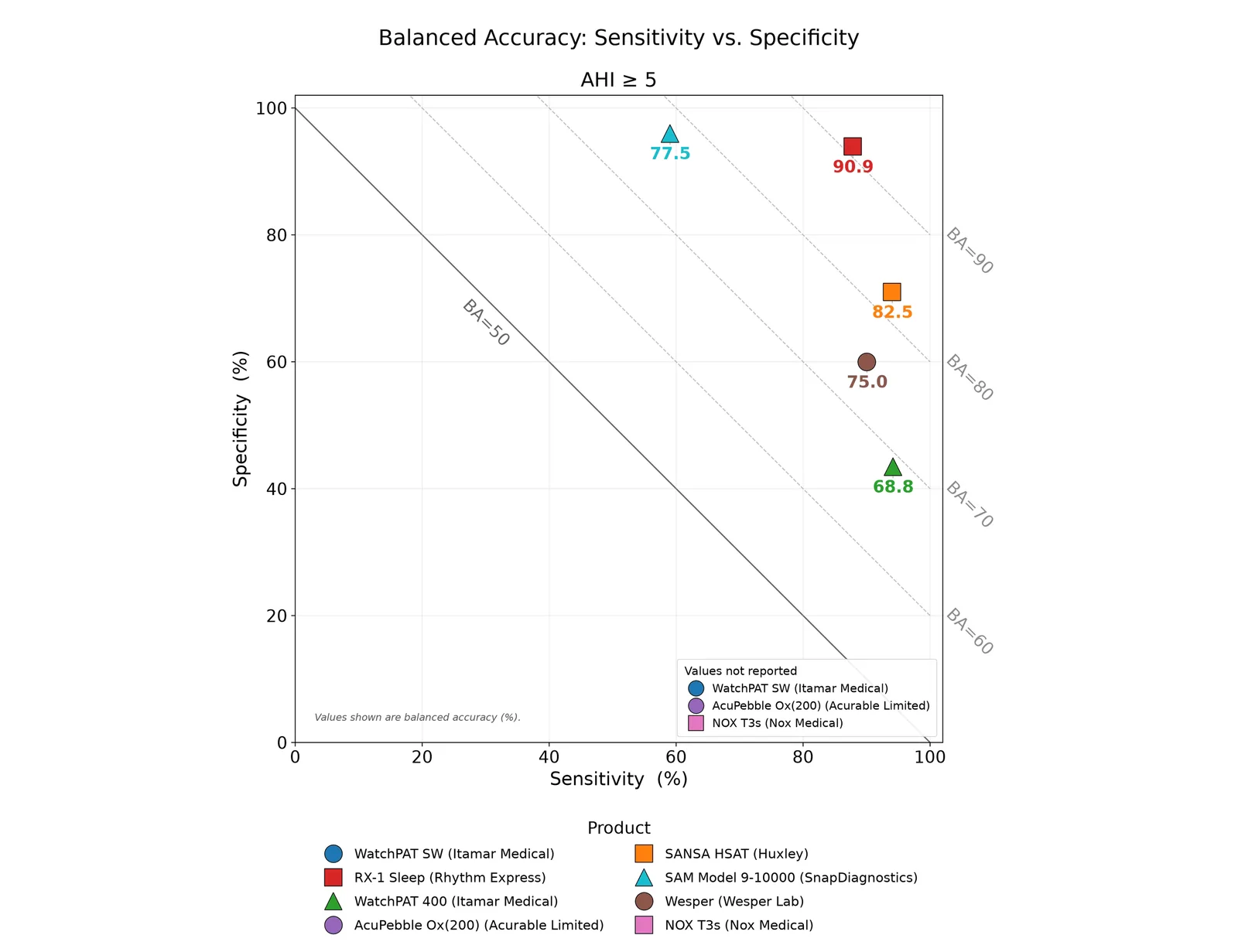
Balanced accuracy of sensitivity and specificity at AHI ≥ 5 (mild to moderate to severe sleep apnea) for the FDA-cleared Type III HSAT devices included in this review Data are shown for the five devices in the comparison set that report performance at this threshold. Devices reported for information only are not plotted AHI: apnea-hypopnea index; FDA: the Food and Drug Administration; HSAT: home sleep apnea testing

**Figure 2:**
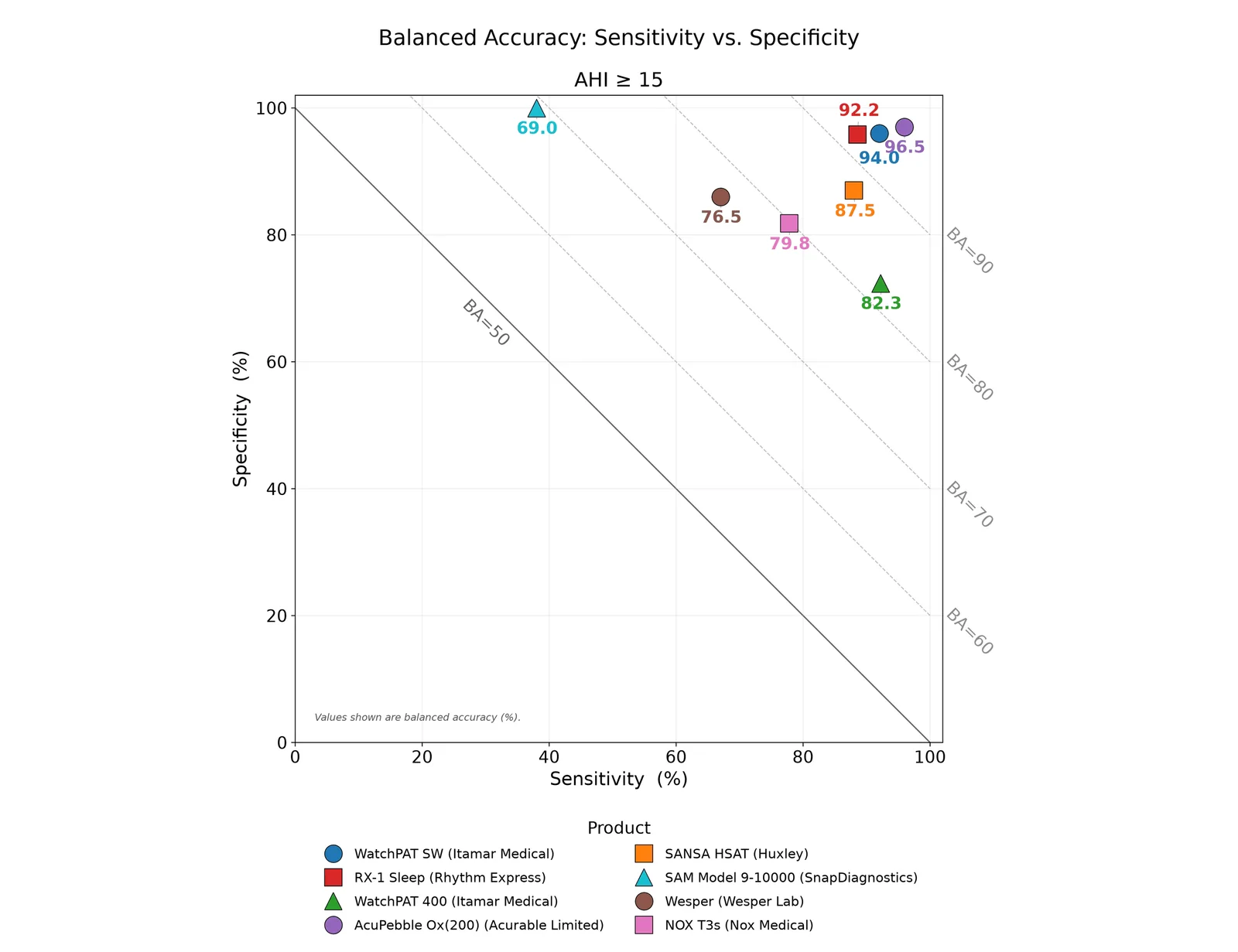
Balanced accuracy of sensitivity and specificity at AHI ≥ 15 (moderate to severe sleep apnea) for the FDA-cleared Type III HSAT devices included in this review Data are shown for the eight devices in the comparison set reporting performance at this threshold. Devices reported for information only are not plotted AHI: apnea-hypopnea index; FDA: the Food and Drug Administration; HSAT: home sleep apnea testing

**Figure 3:**
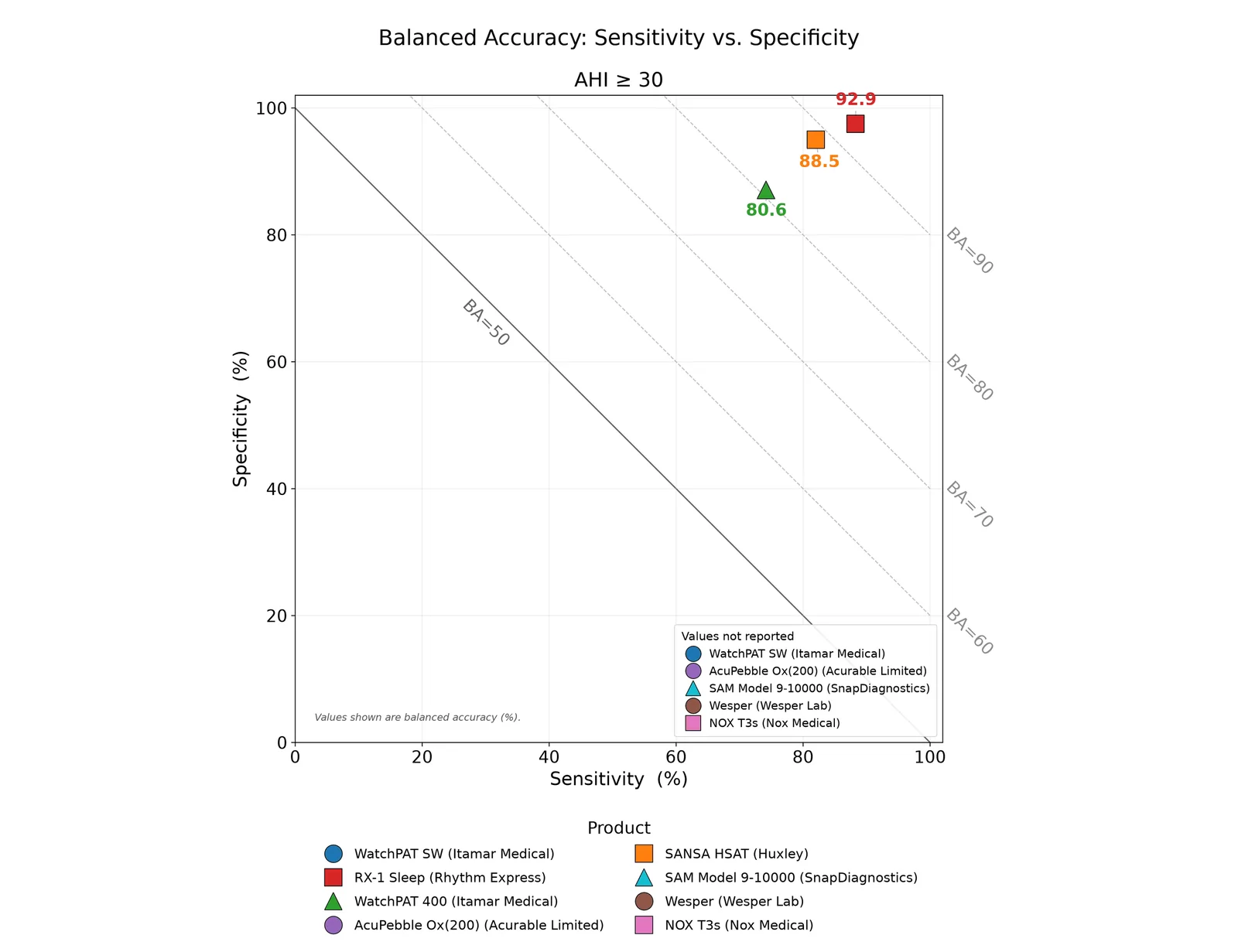
Balanced accuracy of sensitivity and specificity at AHI ≥ 30 (severe sleep apnea) for the FDA-cleared Type III HSAT devices included in this review Data are shown for the three devices in the comparison set reporting performance at this threshold. Devices reported for information only are not plotted AHI: apnea-hypopnea index; FDA: the Food and Drug Administration; HSAT: home sleep apnea testing

These figures should be read with the heterogeneity of their sources in mind. They derive from studies differing in design, population, and size - from 30 subjects for NOX T3s to 476 for RX-1 Sleep - and, in the case of WatchPAT 400, from estimates pooled across multiple clinical studies rather than measured in a single cohort. Confidence intervals were reported for only two of the eight, and no statistical test of between-device difference was performed. A difference of a few points in balanced accuracy, particularly where sample sizes are small or unreported, does not establish that one device outperforms another. What the comparison does show is that the range of published accuracy across currently marketed Type III devices is considerably wider than the shared regulatory pathway would suggest, and that FDA clearance alone is not a reliable indicator of comparable accuracy at the threshold relevant to a given patient.

Multi-night Assessment of Sleep Apnea Severity

Single-night testing assumes that one night’s AHI represents a patient’s usual disease severity. Often it does not. AHI varies substantially from night to night, and severity classification based on a single night has been reported to misclassify a meaningful proportion of patients relative to classification across multiple nights [25,44]. Misclassification matters most near a diagnostic threshold, where an atypical night - one affected by alcohol, sleep position, or short total sleep time - can move a patient across the boundary that determines treatment eligibility or triggers repeat testing [13].

The reviewed devices differ in how many nights they are labeled to record. RX-1 Sleep is used to aid in the assessment of SDB across up to three nights, and RX-1 mini is used for assessment of cardiac rhythm for up to 30 days; NOX T3s is labeled for up to three nights; SANSA HSAT for up to two nights of approximately 10 hours each; and WatchPAT 400 is used for single-night studies. Maximum study duration is not specified in the labeling for the remaining devices. No study identified in this review compared severity classification obtained from multi-night recording with that obtained from a single night using the same device, so this is a difference in labeled capability rather than in demonstrated classification accuracy. Prospective work would be needed to establish that multi-night recording with any of these devices measurably reduces misclassification.

Cardiac Rhythm and Atrial Fibrillation Burden

SDB and atrial fibrillation co-occur frequently, and the association is severity-dependent: among patients with AF, the risk of experiencing at least one hour of AF was 2.3-fold higher on nights of highest SDB severity than on nights of lowest severity [25]. OSA is also associated with an increased risk of major adverse cardiovascular and neurologic events among patients with pre-existing AF [20,21]. A test that characterized both conditions in the same recording would allow that relationship to be observed directly in an individual patient rather than inferred from separate studies.

Labeled capability for this differs sharply across the devices reviewed, as set out in the Results section. Only RX-1 Sleep, used with the RX-1 mini (K241179) [45], aids in the assessment of AF burden alongside SDB. AF burden is reported in every RX-1 Sleep report. SANSA HSAT is cleared to record and store ECG for later manual analysis, but for a maximum of two nights of approximately 10 hours, which is too short to characterize burden. WatchPAT 400 can flag suspected AF and premature beats but is not diagnostic [42], and NOX T3s can detect AF only when an optional ECG channel is added and activated, again without a diagnostic claim [43]. The remaining devices do not record ECG. This is a comparison of what the devices are cleared to do rather than of how accurately they do it: no study identified in this review compared AF detection or burden quantification across these devices, and no clinical outcome data establish that concurrent assessment changes management.

Patient Burden and Recording Quality

Performance as reported in a validation study presumes a technically adequate recording. In practice, sensor discomfort contributes to incomplete and failed studies, and a prematurely terminated recording compromises the full night needed for an accurate AHI [5,8]. The reviewed devices impose markedly different sensor burdens, ranging from systems requiring a chest effort belt and nasal cannula to wrist-worn, finger-worn, and adhesive-patch designs that acquire no direct airflow signal. This is the central design tension in Type III HSAT: the signals that most directly characterize respiratory events are also the ones patients find hardest to tolerate unsupervised. This review did not measure comfort, adherence, or study-failure rates, and those outcomes are not reported consistently across the sources used, so the trade-off could not be quantified. It is a reasonable subject for prospective comparison, and one on which the current evidence base is silent.

Limitations

Because this comparison relied on a mixture of FDA 510(k) Premarket Notifications and validation studies, study design could not be verified, and the recording conditions under which each device’s performance data were obtained - including study duration - could not be confirmed. These data were accepted for FDA clearance; however, substantial equivalence for regulatory purposes does not establish that the underlying studies are comparable for the purpose of a numerical performance ranking, and this analysis should be interpreted with that distinction in mind. Sample sizes for the cited sources ranged from 30 to 476 subjects, could not be confirmed for WatchPAT SW, and are not attributable for WatchPAT 400, whose values are pooled across multiple clinical studies rather than measured in a single cohort. Confidence intervals were available for only four of the 13 entries in Table 1, and no formal statistical test of superiority between devices was performed. The most important constraint is the sparseness of the evidence at the thresholds that matter clinically: of the eight devices in the comparison, all eight report performance at AHI ≥ 15, five at AHI ≥ 5, and three at AHI ≥ 30. Readers should weigh the strength of the evidence behind each device’s reported performance, rather than the point estimates alone, when interpreting Table 1 and Figures 1-3.

Performance estimates compared here were each derived from separate studies in which a single HSAT device was evaluated against PSG; to our knowledge, no published study has directly compared these devices with one another in the same patients. The comparisons presented in Table 1 and Figures 1-3 are therefore indirect, anchored to a common reference standard rather than to one another, and apparent differences between devices may reflect differences in study population, recording conditions, and scoring practice in addition to any true difference in device performance. A direct head-to-head evaluation would require each participant to undergo simultaneous overnight recording with PSG and two or more of the HSAT devices on the same night. Such a protocol raises practical concerns regarding patient tolerance and compliance, competing sensor placement and potential interference between recording systems, and the possibility that the combined sensor burden alters sleep itself and therefore the measurement being made.

These feasibility constraints, rather than any assumption regarding relative device performance, account for the absence of such data. It is relevant in this context that all devices evaluated here were cleared by FDA under the same product code (MNR) and are subject to the same FDA general and special controls, and that each was required to demonstrate acceptable published performance relative to PSG as part of its clearance. This shared regulatory framework provides a common baseline of demonstrated performance across the devices compared. It does not, however, standardize study population, protocol, or scoring methodology, and it should not be interpreted as establishing equivalence or non-equivalence between devices in the absence of direct comparison.

## Conclusions

Across the 13 devices reviewed, reported performance varied widely, and the strength of the evidence supporting it varied more: five devices could be reported only for information, because their published estimates were not obtained on a comparable basis. Among the eight devices that could be compared, only the AHI ≥ 15 threshold was supported by all of them. There, AcuPebble Ox200 reported the highest balanced accuracy, followed by WatchPAT SW and RX-1 Sleep. Coverage is reduced at the other two thresholds: five devices reported data at AHI ≥ 5, and only three at AHI ≥ 30. RX-1 Sleep reported the highest balanced accuracy at both. Comparative evidence at other thresholds, particularly AHI ≥ 30 events/hour, remains limited despite the greater disease severity and clearer indications for treatment. Among the devices compared, only RX-1 Sleep used with the RX-1 mini may aid in the assessment of SDB and AF burden concurrently in a single test, which may allow clinicians to classify OSA severity during remote cardiac monitoring within one home test. As testing for SDB continues to shift toward home-based approaches, confirming the relative performance and clinical value of these devices will require prospective, adequately powered studies that evaluate them head-to-head against a common reference standard with formal statistical comparison, and that determine whether multi-night testing and AF burden detection change treatment decisions and cardiovascular outcomes.
